# Plant Cytogenetics in the Micronuclei Investigation—The Past, Current Status, and Perspectives

**DOI:** 10.3390/ijms23031306

**Published:** 2022-01-24

**Authors:** Jolanta Kwasniewska, Adrianna Wiktoria Bara

**Affiliations:** Plant Cytogenetics and Molecular Biology Group, Faculty of Natural Sciences, University of Silesia in Katowice, Jagiellonska 28, 40-032 Katowice, Poland; adriannabara@gmail.com

**Keywords:** chromosome aberrations, cytogenetics, DNA damage, micronuclei, mutagenesis

## Abstract

Cytogenetic approaches play an essential role as a quick evaluation of the first genetic effects after mutagenic treatment. Although labor-intensive and time-consuming, they are essential for the analyses of cytotoxic and genotoxic effects in mutagenesis and environmental monitoring. Over the years, conventional cytogenetic analyses were a part of routine laboratory testing in plant genotoxicity. Among the methods that are used to study genotoxicity in plants, the micronucleus test particularly represents a significant force. Currently, cytogenetic techniques go beyond the simple detection of chromosome aberrations. The intensive development of molecular biology and the significantly improved microscopic visualization and evaluation methods constituted significant support to traditional cytogenetics. Over the past years, distinct approaches have allowed an understanding the mechanisms of formation, structure, and genetic activity of the micronuclei. Although there are many studies on this topic in humans and animals, knowledge in plants is significantly limited. This article provides a comprehensive overview of the current knowledge on micronuclei characteristics in plants. We pay particular attention to how the recent contemporary achievements have influenced the understanding of micronuclei in plant cells. Together with the current progress, we present the latest applications of the micronucleus test in mutagenesis and assess the state of the environment.

## 1. Introduction 

Cytogenetics is the branch of genetics, cytology, and cell biology that analyses the nuclear genomes at the chromosome level. Cytogenetics makes the chromosome a substantial target in elementary plant cell biology and other fields such as mutagenesis and genotoxicity studies. Standard cytogenetic methods were, and are still, commonly used. Modern cytogenetic technologies involving advanced microscopy and imaging methods, that progress in the analyses on epigenetic DNA and histone modifications as well as DNA damage by using fluorescent antibodies benefit plant genome structure, dynamics, and evolution. They have also served the comprehensive evaluation of the effects of various mutagens on the plant genome that are observed as chromosome aberrations, including micronuclei (MN). Mutagens affect the structure of DNA and cause double-strand breaks (DSBs) leading to MN formation. The elimination of MN causes DNA loss. Micronuclei are induced by many mutagenic factors, both physical and chemical, as well as those of an environmental nature. The analysis of their frequency is the basis of the commonly used micronucleus test. We provide a comprehensive overview of the current knowledge on MN characteristics in plants. This paper focuses on critical scientific problems: Is the distribution of DNA damage that led to micronuclei formation random? What is the origin of plant micronuclei? Are epigenetic processes involved in micronuclei formation? How could there be a role of the genetic activity of chromatin in the formation of micronuclei?

## 2. The Importance of the Micronucleus Assay in Plants

Micronuclei (MN) are structural chromosome aberrations that are detected in non-dividing cells during interphase. Among numerous genotoxicity assays, the micronucleus (MN) test is especially recommended to evaluate the genotoxic effects of chemical and physical agents, as well as mixtures of substances. Since 1959 when the MN assay was first applied in kidney beans, followed the treatment with gamma-ray [1], it served as a well-established, fast, and reliable routine system for measuring the genome damage that is caused by genotoxic agents in mitotic and meiotic plant cells [2,3]. Currently, the MN test is still successfully used in testing many agents, including pesticides, nitroaromatic compounds, polyaromatic hydrocarbons, nitrosamines, heavy metals, ionizing radiation, and industrial chemicals, as well as other environmental samples [4,5,6]. Nowadays, the interactions of nanoparticles with plants have become a new field in micronuclei assays [7,8,9]. Currently, the testing is mainly performed in *Allium*, *Nicotiana*, and *Vicia* [10,11,12,13] and other model plants [7,14,15,16,17].

Compared to the chromosomal aberrations (CA) assays that are applied to mitotically divided cells, the MN test is less time-consuming and easier to perform. Most mutagens decrease mitotic activity, thus making the chromosome aberrations analyses in dividing cells, especially in metaphases, challenging and often impossible. 

Although the knowledge on different aspects of the origin, structure, genetic activity, and micronuclei in plants has been explored in recent years, there is still much less that is known than in humans and animals. There are many reviews of MN in humans and animals, also from the last few years [18,19,20,21,22,23,24,25,26,27]. Micronuclei have become a potential linkage biomarker to cancer and aging-related diseases [28]. The MN test is now quite widely described in plants [29], although there are still no reviews that summarize all the data on MN, with particular reference to the latest methodological developments in the field of molecular cytogenetics. 

## 3. Micronuclei—The Formation and Fate

Micronuclei (MN) are small, extranuclear bodies that are located next to the parental nucleus in the cytoplasm. Micronuclei are detected in the meristematic interphase cells of the shoots or roots, in the next cell cycle, followed by treatment with mutagen. 

MN can originate in two ways. They can arise from acentric fragments resulting from double-strand breaks (DSBs) which are not repaired or repaired improperly. The micronuclei could also occur from the entire chromosome(s) that does not attach to the mitotic spindle at metaphase. Likewise, micronuclei that have arisen from entire chromosomes could result from kinetochore damage, failure of the cell cycle control system, or centromeric DNA hypomethylation. Thus, the knowledge on the origin of micronuclei allows for assessing the mutagen’s mechanism of action as clastogenic or aneugenic. Changes in the structure of the chromosomes, such as chromosome fragments and delayed chromosomes, can also be detected during mitosis; however, their detection is not as straightforward as during interphase. Some micronuclei might also be derived from the breakage of anaphase bridges that are formed from dicentric chromosomes, concatenated ring chromosomes, the union of sister chromatids, unresolved sister chromatid connections, or chromosomes that have merged by telomere fusion. 

The number of micronuclei in a single cell is most often one, but sometimes cells with a higher MN number are observed, depending on the number of chromosome fragments or delayed chromosomes. It still needs to be emphasized that, taking into account the mechanism of micronucleus formation, the frequency of dividing cells after the mutagenic treatment influences the frequency of micronuclei. Cells need to divide so that chromosome fragments can be removed outside the newly formed daughter nuclei and create an MN.

From the data that are available for animals and humans, micronuclei can be lost from the cells and incorporated into the nucleus [30]. There are no specific data on the fate of micronuclei in plants cells. 

## 4. Conventional Cytogenetics 

**Figure 1:**
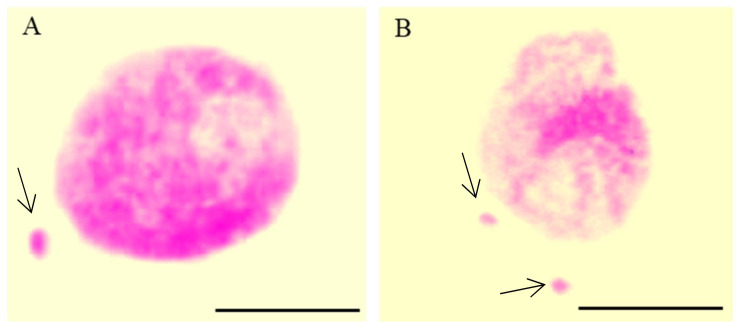
Nuclei with micronuclei (MN): one MN (**A**) and two MN in one cell (**B**) after maleic acid hydrazide (MH)-treatment in *Crepis capillaris* root meristematic cells; Feulgen technique. Arrows show the micronuclei. The bars represent 5 µm.

The conventional cytogenetic is recognized as the approach for the detection and basic description of the MN after mutagenic treatment. Changes in chromosome morphology are usually detected using the basic chromosome-staining techniques, such as the Feulgen method, acetoorcein, and Giemsa stainings. Among these staining methods, the Feulgen technique (Figure 1) is characterized by the best contrast of chromatin staining; however, this procedure needs a longer time and a more complicated process. Together with slide scoring, these techniques allow the analysis of the frequency of MN. Possibly too small MN are not detected with these methods, and only those that arise from whole chromosomes or large chromosome fragments are visible. 

**Figure 2:**
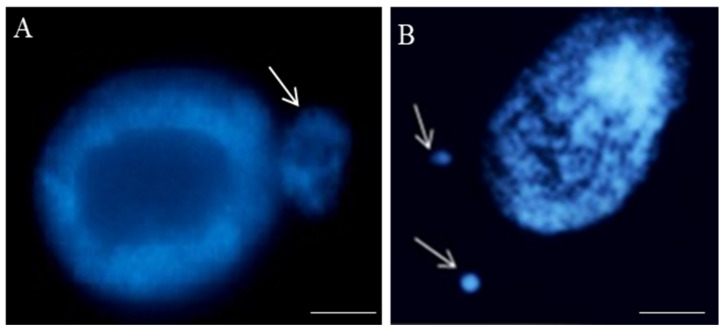
Nuclei with micronuclei: one (**A**) and two in one cell (**B**) after maleic acid hydrazide (MH)-treatment in the root cells of *Crepis capillaris* seedlings. The micronuclei differ in size; DAPI staining. Arrows show the micronuclei. The bars represent 20 µm.

Nowadays, fluorescent methods, e.g., DAPI (4′,6-diamidino-2-phenylindole staining) (Figure 2) or acridine orange stainings rather than traditional methods, are recommended for micronuclei detection and scoring instead of conventional methods. 

The fluorescence methods are quick and precise, and even small micronuclei can be detected [31]. Still, the analysis of micronuclei using these simple methods does not provide any information on the localization of the DNA breaks and the mechanisms that lead to their formation.

## 5. Molecular Cytogenetics 

The early and current achievements of molecular cytogenetics have led to progress in the detection and detailed characterization of micronuclei (MN) [32,33]. Modern cytogenetics techniques have revolutionized knowledge on the composition and genetic activity of the chromatin that is involved in micronuclei. The knowledge on the specific genetic content of the micronuclei is essential as they could be related to the ability of chromatin in the micronuclei to exert proper DNA expression and DNA repair. Among techniques, fluorescence in situ hybridization (FISH) and all its modifications have been successfully used in the modern generation era of DNA damage characterization. Molecular cytogenetics that is based on the multi-fluorescence detection of the specific chromosomes landmarks or painting whole chromosomes represents a milestone in DNA damage analyses in relation to genome organization. Additionally, it enables the studies of even minute details of the chromosome, providing the analyses of DNA damage more accurately and precisely. In the time of sequencing of plant genomes, FISH becomes even more important as many new chromosome-specific probes become available. Moreover, the cytogenetic analyses of DNA and histone epigenetic modifications on plant chromosomes and nuclei provide new possibilities to learn the role of plant chromatin dynamics in response to mutagens [34]. Currently, the involvement of the histone modifications was proven to be closely related to plant environmental stress [35]. 

### 5.1. Fluorescence In Situ Hybridization Serves to Understand the Origin of Micronuclei

A breakthrough in the analyses of the localization of DNA damage at the chromosomal level in plants came with applying the fluorescence in situ hybridization (FISH). It provides information on the possible ‘hot spots’ in plant genomes for DNA damage after the action of mutagens. Also, it gives information on the mechanisms of the biological effect of the individual agents that induce DNA damage. This knowledge is particularly crucial in plant mutagenesis, as the use of the chemical and physical mutagens is the most common way to obtain mutants. This technique could detect even extremely small aberrations in dividing and non-dividing cells. 

There is only one morphological type of micronuclei that may differ in size (Figure 2). The size of the micronucleus does not provide any information on whether it originated from chromosome fragments or entire chromosome(s), as the size may be related to the different degrees of the chromatin condensation. A more detailed analysis of the involvement of a specific chromosome or chromosome fragments in micronuclei formation is possible using fluorescence in situ hybridization (FISH). So far, FISH has not found such a wide application in the study of chromosome aberrations, including MN, in plants, as it has in humans [36,37,38]. Different types of DNA probes for FISH are applied in plants, e.g., repetitive DNA sequences, single-locus chromosome-specific BAC clones, partial (e.g., arm), and whole chromosome paints. The limitations of the chromosome-specific DNA sequences in plants make the comprehensive identification of chromosome fragments in micronuclei using FISH still limited to a few species. Among the repetitive DNA sequences, centromere, *Arabidopsis thaliana* (Arabidopsis)-type (TTTAGGG)n telomeric sequences, and ribosomal DNA (rDNA), which give strong and easily observed FISH signals, have found application in the detailed characterization of MN. These DNA sequences’ advantages are evolutionary conservation and location at a specific chromosome region. Repetitive dispersed DNA sequences are not a good source for probe pool for fluorescence in situ hybridization to study the origin of micronuclei.

**Figure 3:**
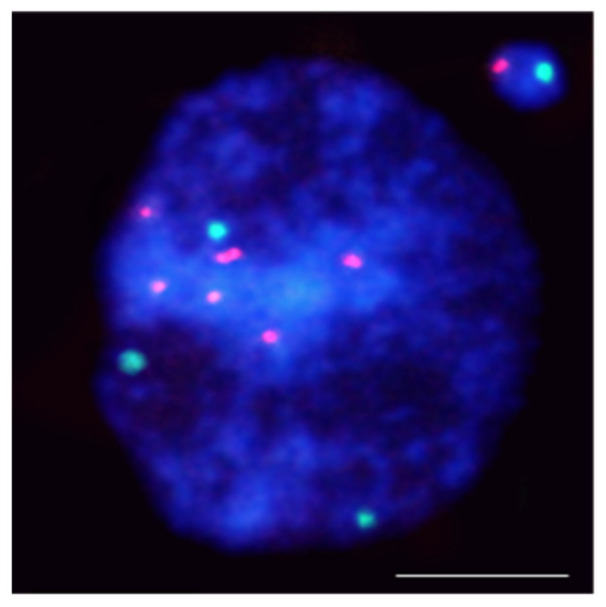
*Hordeum vulgare* interphase nuclei with the micronucleus induced by X-radiation. The nucleus was subjected to mcFISH with 5S rDNA (red) and 25S rDNA (green) probes. The micronucleus has one 5S rDNA and one 25S rDNA. Chromatin is stained with DAPI (blue). The bar represents = 10 µm.

FISH using 45S rDNA as the probe was first applied to localize the chromatin aberrations, such as translocations [39] and anaphase bridges [40], in *Arabidopsis thaliana*. Applying the rDNA as probes showed rules regarding gamma-ray–induced MN formation in barley (*Hordeum vulgare*) (Figure 3). 

5S rDNA-bearing chromosomes are shown to be more often involved in MN formation than NOR chromosomes in barley [41,42]. Similar rules regarding radiation-induced MN formation have been found in *Brachypodium distachyon* [43]. The hot spots for chromosome breakage in *Lolium multiflorum* were not correlated with rDNA sites [44].

**Figure 4:**
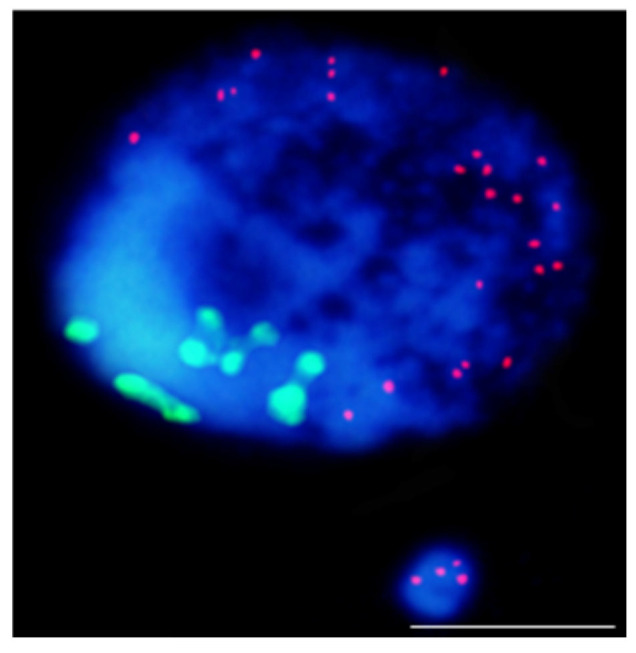
Results of mcFISH with telomeric (red) and centromeric (green) probes. *Brachypodium distachyon* interphase nuclei with micronucleus that were induced by X-radiation; micronucleus shows only telomeric DNA signals. The bar represents 5 µm. Micrograph by A. Kus.

The use of the centromere and telomere-specific DNA sequences for FISH also provided some rules regarding the origin of MN. It confirmed that the gamma ray-induced MN may originate from acentric fragments or whole lagging chromosomes. Thus, this approach allows the distinguishing of the micronuclei being a clastogenic and aneugenic effect of mutagens. However, most MN had only telomeric DNA signals, indicating that terminal deletion is the primary type of chromosome aberration leading to their formation (Figure 4). 

Comparing the contribution of particular chromosome fragments in MN that are induced by different chemical clastogens, the maleic acid hydrazide (MH) and nitroso-N-methyl-urea (MNU) have shown the difference in the size of the chromosome fragments that are involved in the MN. Most MH-induced MN originated from large acentric fragments, whereas MNU-induced MN is from small terminal chromosome fragments [41,42]. 

FISH provides much more information about MN formation with DNA probes that are dedicated to different chromosomes or particular chromosomes. Standard A- and B-chromosome-specific probes were successfully used in the rye gamma-irradiated cells (*Secale cereale* L.) [45] for the detection of the translocations between the A- and B-chromosomes.

One of the FISH approaches that is used to detect and characterize micronuclei in plants is multicolor FISH (mcFISH). It is based on the two consecutive FISH experiments that use a pair or pairs of probe sets that are removed after each experiment and include the reprobing step. Combining more than two differently labeled DNA probes on the same nuclei slide makes this technique more informative [46]. For the first time, mcFISH has been applied in human carcinogenicity studies [47], then it has found application in mammalian cells [48]. mcFISH is a common technique that is widely used in plants; however, it has narrow application in plant mutagenesis and genotoxicity. For the first time, this approach was applied in the analysis of the involvement of four different DNA sequences: 5S rDNA, 25S rDNA, the Arabidopsis-type (TTTAGGG)n telomeric sequence, and the Brachypodium-originated centromeric BAC clone CB33J12 in the micronuclei formation in *Brachypodium distachyon* root-tip cells that were subjected to a chemical mutagen [43]. 

**Figure 5:**
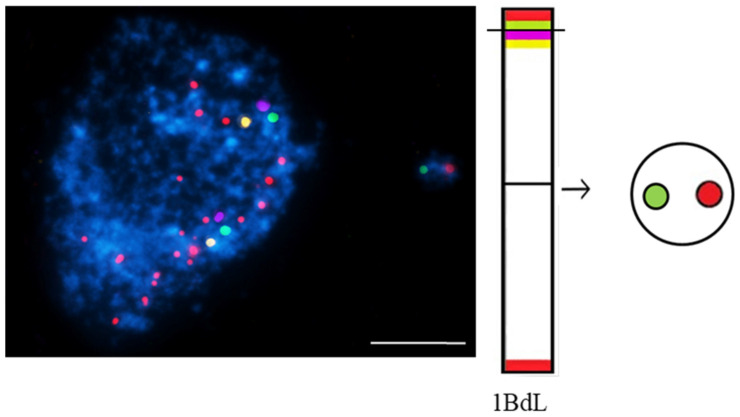
*Brachypodium distachyon* interphase nuclei with micronuclei that were induced by MH-treatment that were subjected to mcFISH with the following probes: telomeric sequence (red), I BAC pool (green), II BAC pool (violet), and III BAC pool (yellow). Chromatin is stained with DAPI (blue). The diagram next to the photomicrographs shows the putative origins of the micronuclei. Transverse dashed lines indicate chromosome breakpoint. The scale bar = 5 µm. Micrographs by A. Kus.

The most advanced FISH-based approach in plants is chromosome painting (CP), which permits the selective visualization of entire chromosomes or their specific segments during mitosis as well the interphase [49,50,51,52,53,54]. The wide use of this technique for humans and mammals to determine the involvement of specific chromosomes in the formation of micronuclei showed that they preferentially comprise particular chromosomes that are related to the chromatin organization [55]. The large amounts of repetitive DNA on all chromosomes are obstacles to CP on plants. CP is limited to a few plant species: Arabidopsis [56], Brachypodium [57], and few other species that are characterized by a small genome. mcFISH and CP with low repeat (small and large pools of bacterial artificial chromosomes (BAC)) clones that are specific for selected chromosomes, were applied to improve the ‘standard’ MN test in *Brachypodium distachyon* (Figure 5). 

BAC-FISH-based chromosome painting provides new information on the composition, origin, and mechanisms of micronuclei formation that is induced by MH-treatment and X-radiation in Brachypodium by showing the ‘fragile spots’ of DNA breaks [58]. Site-specific DNA breaks in chromosomes Bd4 and Bd5 were shown [59]. 

To summarize, FISH provides new insights into the localization of DNA breaks on plant chromosomes, proving the non-random distributions of chromosome aberrations. The reasons for this non-random distribution may be the spatial organization of the nucleus at the interphase, the diverse transcriptional activity of specific chromosome regions, and chromosome size. Single BAC-FISH-based chromosome barcoding and ‘chromosome painting’ approaches have proven to be effective in analyzing the mechanism of micronuclei formation in plants after mutagenic treatment. The advantages of the FISH technique in terms of accuracy and quality of quantitative analyses make the technique one that is likely to become more widespread in DNA damage studies in plants.

### 5.2. Genetic Activity and DNA Replication 

The nucleolus, whose primary function is ribosomal RNA (rRNA) synthesis and ribosome biogenesis, plays a crucial role in the response to biotic and abiotic stress. This aspect has not been extensively studied in plants [60]. Various stresses can lead to alterations in the protein content and organization of plant nucleoli due to alterations in nucleolar transcriptional activity [61]. The nucleolus, including rRNA genes that are arranged in tandem DNA arrays, is observed during interphase. Then nucleoli are reconstituted on NOR sites during mitosis. Its activity differs depending on the environmental conditions [62].

The p53 transcription factor plays a significant role in the DNA damage response (DDR) in mammalian cells to maintain genome stability [63]. Plants developed their unique system for stress response that involved nucleolar proteins; many plant proteins are involved in DDR [64,65]. 

**Figure 6:**
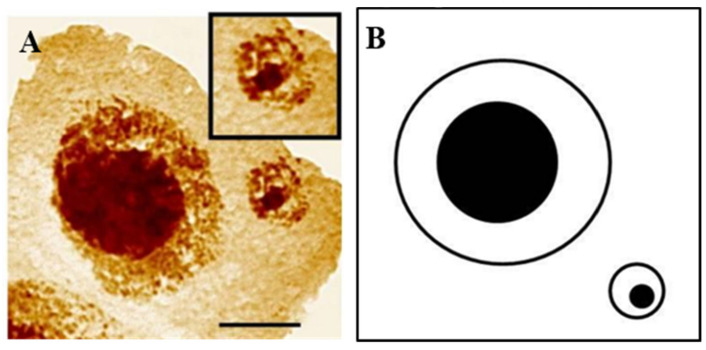
*Hordeum vulgare* interphase cell with micronucleus after treatment with MH. Staining with the silver-staining method (**A**) and scheme (**B**). The bar represents 10 μm. Micrographs by J. Jaskowiak.

In plants, cytogenetic studies of the activity of rRNA genes in MN seem to be particularly important as the frequent involvement of the rRNA genes in their formation was shown for a few species: barley (Figure 6), Brachypodium, and *Crepis capillaris* [42,43]. 

The transcriptional activity of 35S rRNA genes that are present in MN that were analyzed using silver-staining is always maintained in barley [66]. MN in *Vicia faba*, with a nucleolar organizer, could synthesize protein and replicate DNA [67]. Studies on the transcriptional activity in plants in the main nuclei after being subjected to different stresses are more common than in the micronuclei. The changes in the number and size of nucleoli, their disintegration, and leakage into cytoplasm were detected in plant cells in response to various stress factors [68,69]. The molecular aspects of nucleolar stress responses in plants were reviewed by Ohbayashi et al. [70]. 

Many studies on the transcriptional activity in micronuclei were performed in humans [71]. It depends on the micronuclear content; the micronuclei that originate from the whole chromosome show transcriptional activity, whereas MN containing acentric fragments do not. The role of nuclear pore complexes is being considered in cancer cells [72]. 

Precise genome replication is crucial in maintaining the stability of genomes and any replication errors are critical for living cells. The studies on the genetic activity of chromatin in MN also includes the ability to replicate DNA. The early studies on the micronuclear chromatin replication indicate the heterogenous behavior of MN in animal cells [73]. DNA synthesis was studied using pulse labeling of cells with bromodeoxyuridine (BrdUrd, BrdU) followed by the immunofluorescence detection with anti-BrdUrd antibodies. If the micronuclear DNA can replicate, it also usually occurs in the main nucleus. DNA synthesis in micronuclei corresponds with nuclei during the S-phase in approximately 98% of the micronuclei.

**Figure 7:**
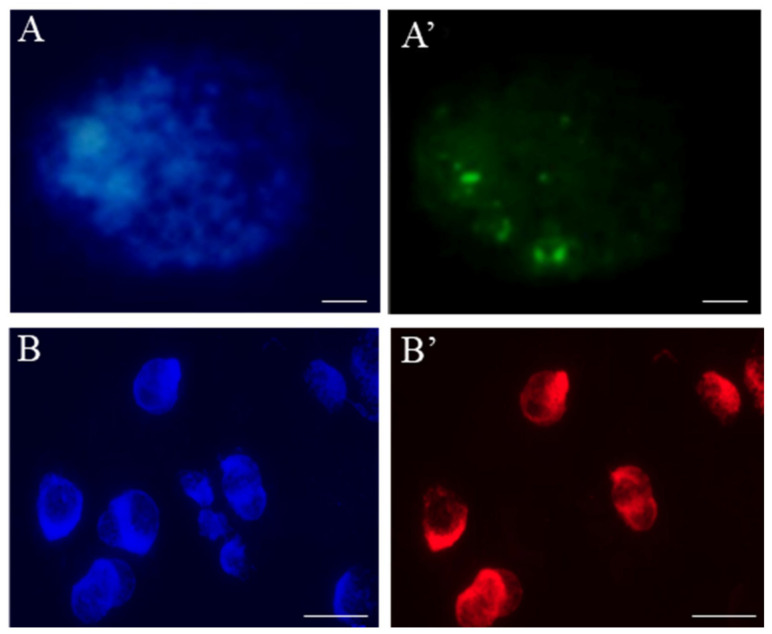
Localization of the replication sites in *Hordeum vulgare* root-tip nuclei using (**A’**) EdU and (**B’**) BrdU labelling. Nuclei are counterstained with DAPI (**A**,**B**). The bars represent 5 µm.

Nowadays, there has been progress in detecting S-phase nuclei and DNA replication in the MN. BrdU, with many disadvantages, such as a denaturation step and low specify that is correlated with the size of antibody signals, has been replaced by modern labeling higher resolutions techniques—“click” reaction with 5-ethynyl-2′-deoxyuridine (EdU) [74,75] (Figure 7).

Distinct rules have been observed in plant cells when the replication ability of micronuclei was analyzed using the pulse EdU labeling method [76]. The presence of S-phase labeling characterized only 1% of the micronuclei. The ability of micronuclear chromatin to be replicated is greatly influenced by the specific genetic content of the micronucleus. 

### 5.3. DNA Damage and Repair 

The DNA damage response (DDR) plays a role in maintaining the genome integrity in response to abiotic and biotic stresses [77,78,79,80,81]. The final effect of a mutagenic treatment is the primary DNA damage and the process of DNA repair. Strand breaks, which can lead to changes in the chromosome structure, including MN, are the most important types of damage that have been observed at the DNA level. Of the 5000 single DNA breaks that were generated during one cell cycle, only 1% are converted into double DNA breaks (dsDNA); micronuclei constitute a significant result of dsDNA. 

Many methods have been developed to detect and localize DNA damage in a genome, quantify the repair processes, and thus provide better insight into the mutagenesis process in various organisms [82]. DNA breakage after mutagenic treatment can be quickly evaluated using the TUNEL (terminal deoxynucleotidyl transferase-mediated dUTP nick-end labeling) test to analyze the frequencies of cells that have fragmented DNA [76]. It detects single and double DNA strand breaks in interphase nuclei. The 3′-OH termini are enzymatically-labeled with a modified nucleotide such as fluorescein dUTP. The reaction is catalyzed by the terminal deoxynucleotidyl transferase (TdT), and the signals are detected using fluorescence microscopy. All the nuclei are simultaneously stained with another fluorochrome, e.g., DAPI (4′,6-Diamidino-2-phenylindole), and, therefore, the percentage of damaged nuclei using positive labeling is possible. The advantages of the TUNEL test are its ability to detect DNA breaks in a single nucleus with the possible analysis of specific localization within it as well as the short time that is required for an assay and the easy screening of the labeled nuclei. This test has been recommended for the preliminary evaluation of the genotoxicity of any newly tested agent, both in the main nuclei and the micronucleus. 

### 5.4. Chromatin Structure and Its Role in Response to Mutagens

Epigenetic modifications of chromatin, which are defined as being mitotically- and meiotically-heritable changes in the gene expression patterns that arise independent of the changes in DNA sequence, are essential for many biological processes, including growth and reproduction. Post-translational modifications of histones and DNA methylation are the main epigenetic modifications that have a causal role in establishing different chromatin states. Chromatin is a dynamic complex of DNA and proteins. The two main chromatin states can be distinguished: compacted and repressed, the so-called heterochromatin, or the less condensed and gene-rich euchromatin. Earlier studies indicated that the heterochromatic regions represent ‘hot spots’ for the aberrations that are induced by S-phase-dependent mutagens. 

DNA methylation is one of the epigenetic modifications that has been studied in plants most intensively. At the chemical level, this process involves the covalent addition of a methyl group to the 5th position of cytosine in a pyrimidine ring. It is catalyzed by the methyltransferase enzymes using S-adenosyl methionine as the methyl group donor. In plants, the heterochromatin domains are determined by the methylation of cytosines (5mC), and there is a close link between DNA and histone methylation. DNA methylation is highly concentrated in the heterochromatin domains, mainly in the centromeric regions and repetitive sequences. Cytologically, heterochromatin, which has a high level of methylated DNA, can be defined as intensively DAPI-stained chromocenters during the interphase.

**Figure 8:**
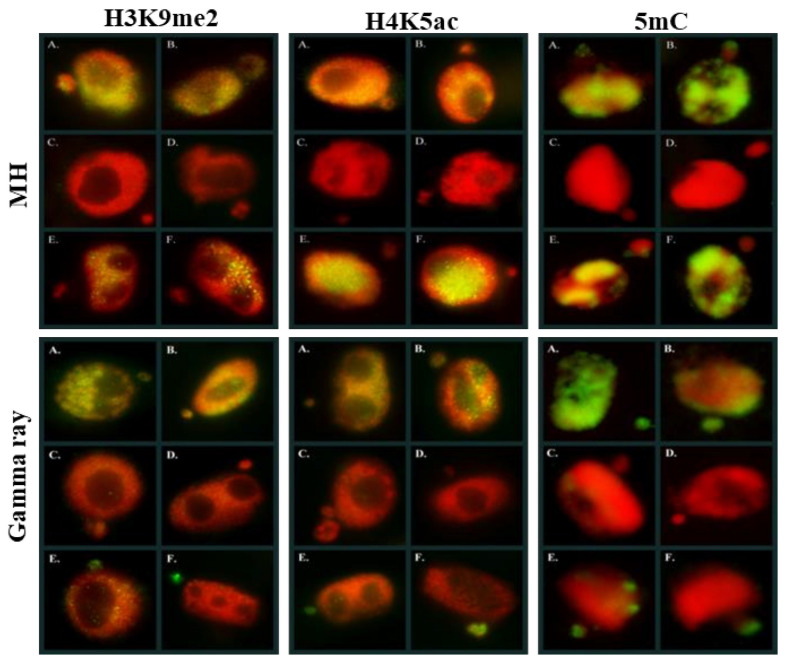
Histone and DNA epigenetic modifications in *Hordeum vulgare* nuclei and micronuclei after MH and gamma rays treatments. DAPI—red, histone-modifications (H3K9me2, H4K5ac) and 5mC—green. Micrographs by A. Braszewska.

Additionally, the same specific patterns of 5mC can be found along the metaphase chromosomes [83]. Also, DNA demethylation occurs in plants. This phenomenon can be achieved through passive DNA demethylation, e.g., during the replication process or active DNA demethylation via the action of specific demethylating enzymes. DNA methylation is involved in the plant’s response to environmental stresses. Recent studies have shown the differential regulation of genes encoding epigenetic regulators and chromatin and DNA methylation changes in response to various abiotic stresses, including cold, salinity, drought, and osmolality [84]. There are some studies on epigenetic modifications’ involvement in the MN formation in mammals, and only single study in plants to date. Based on the studies on humans, it was shown that the MN formation was induced epigenetically mainly through the loss of DNA methylation. Specifically, the hypomethylation of heterochromatin in the pericentromeric regions was associated with chromatin decondensation, which leads to incorrect chromosome segregation and exclusion into the MN [19]. Our previous study on the impact of two mutagenic agents: chemical—maleic acid hydrazide (MH) and physical—gamma rays on the global epigenetic modifications of chromatin H3K9me2, H4K5ac, and 5mC in barley revealed that MN in barley could have a low or high level of specific epigenetic modifications (Figure 8). 

However, similar levels of histone H3 methylation, histone H4 acetylation, and 5mC in the MN and its parental nucleus were observed more often. Rarely, the differences in the level of epigenetic modification between the MN and its parental nuclei were observed [85]. The evaluation of DNA methylation in a single nucleus and micronucleus in *B. distachyon* genome was studied. DNA methylation might respond to mutagenic treatments [86]. It demonstrates that analyses of the epigenetic modifications should be integrated into current plant genetic toxicology and mutagenesis. 

### 5.5. Imaging Approaches 

For the MN test, the microscope is obligatorily used for the visual detection of micronuclei and their manual counting based on observing a significant number of cells. Despite the many advantages of MN, their analysis by picking out and manually counting with microscopy is time-consuming, requires the proper skills, and is prone to subjectivity. On the other hand, the visual confirmation of MN and visualization of cytoplasm to associate MN to a particular cell is an advantage of this method. The use of high definition fluorescence microscopy that is equipped with a high-sensitivity camera allows the precise detection and quantification of micronuclei and automatically captures images. The development of microscopic and bioimaging techniques enables the rapid and versatile assessment of MN. These approaches improved the statistical power of this method and the robustness of the MN assay. Previously, laser scanning cytometry (LSC) and conventional flow cytometry methods were successfully applied to identify and enumerate MN [87,88]. This method was fraught because MN is not correctly distinguished from other DNA bodies, debris, and nuclei [89]. A flow cytometry-based approach testing micronucleus induction (FCMN assay) was also tested for humans to detect nanomaterials-induced MN [90]. Due to many scored cells and the compatibility of the results with other tests, the FCMN approach can serve as a speed assay to evaluate the potential genotoxicity as MN formation. A fully automated Image Stream Imaging Flow Cytometer has been developed to perform the in vitro micronucleus assay [91]. It combines the speed of the high-throughput nature of conventional flow cytometry with the visual information of high-resolution microscopy. Another method, single cell quantitative imaging microscopy (scQuantIM) accurate for quantifying the frequency of micronuclei formation for biomedical research; so far was optimized and tested for cancer cells, treated by genotoxic agents, etoposide, or bleomycin [92]. All the above technical innovations have been developed and used for human research; however, they hopefully can be applied to plant cells in the future.

## 6. Concluding Remarks and Future Perspectives 

In conclusion, we have highlighted the possibilities of the detection and detailed analysis of MN in plants, emphasizing the research directions using modern molecular cytogenetic approaches. These collected approaches provide future directions to study MN in plants. 

The most important advancement from the development of the molecular cytogenetic techniques for the MN analysis is based on fluorescence in situ hybridization (FISH) and its variants. With the application of FISH, many obstacles that are connected to the small size and uniformity of chromosomes were overcome. Thus micronuclei, which are very small, could be analyzed even in species that are characterized by a small genome. The other cytogenetic advances, e.g., in chromosome preparation, such as extended fiber-FISH, are in no way needed for the study of MN, although they are very helpful in the analysis of plant genome structure. The imaging and signal amplification technologies have improved the ability to detect small gene-sized probes in micronuclei. Recently, the main driver of plant cytogenetics are next generation sequencing (NGS) platforms, as well as bioinformatics that enables analyses of DNA sequences. Up to date, based on the whole genome sequencing achievements for many species, the linking DNA sequence to the physical chromosomes enable the development of new areas of plant genomics, epigenetics, and evolution. The integration of the big data and next-/third-generation sequencing, with the cytogenetics offers possibilities for new insight into micronuclei structure in the future.

**Figure 9:**
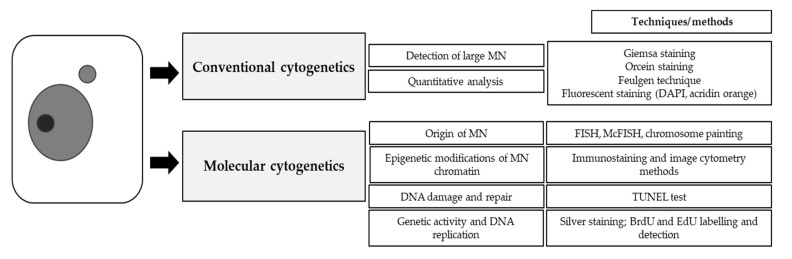
Possibilities of micronuclei assessment in plants.

Although several advances have recently been made in the studies of MN in plants (Figure 9), a number of important questions still need to be addressed, namely whether micronuclei can be re-engulfed by the cell nucleus and whether the micronuclear content can be degraded independently of further cell divisions. 

There is no knowledge on the possible cell lethal events and their risk to the organism. These new approaches may help to clarify whether micronuclei and genomic instability are related to other cellular mechanisms that have not been described so far.
